# Complete Genome Sequence of Oxidase-Positive Stenotrophomonas maltophilia Strain SM7059

**DOI:** 10.1128/MRA.01576-18

**Published:** 2019-04-25

**Authors:** Shuo Gao, Zhifeng Zhang, Hui Zhou, Hong Zhu, Han Shen, Wanqing Zhou

**Affiliations:** a Department of Laboratory Medicine, Nanjing Drum Tower Hospital, the Affiliated Hospital of Nanjing University Medical School, Nanjing, Jiangsu, China; Broad Institute of MIT and Harvard University

## Abstract

Stenotrophomonas maltophilia is an opportunistic pathogen which causes an increasing frequency of infections in hospitalized patients. Here, we present the complete genome sequence of Stenotrophomonas maltophilia SM7059, an oxidase-positive strain isolated from a female patient with hepatolithiasis in China.

## ANNOUNCEMENT

Stenotrophomonas maltophilia is an aerobic Gram-negative bacillus typically found in soil and water, and it is also frequently isolated as an opportunistic pathogen in hospitalized patients (1). Standard microbiology references describe S. maltophilia as oxidase negative, while an analysis of a collection of 766 S. maltophilia isolates indicated that approximately 20% are oxidase positive, and this species should be reevaluated for other phenotypes (2). Here, we present the complete genome sequence of a clinical oxidase-positive S. maltophilia strain, SM7059, which is helpful in understanding the expression of oxidase.

S. maltophilia strain SM7059 was isolated from an ascites sample from a female patient admitted to the hospital for hepatolithiasis in 2017 in China. The isolate was identified as S. maltophilia using the Vitek 2 system (Sysmex-bioMérieux, Marcy-l’Étoile, France). Microbact oxidase strips impregnated with *N*,*N*,*N*′,*N*′-tetramethyl-*p*-phenylene-diamine dihydrochloride (Oxoid Ltd., UK) were used to detect cytochrome oxidase enzyme production in S. maltophilia strain SM7059. Escherichia coli (ATCC 25922) and Pseudomonas aeruginosa (ATCC 27853) were used as a negative control and a positive control, respectively.

Genomic DNA was extracted from a culture grown overnight in Luria-Bertani medium at 35°C under agitation (220 rpm) using the QIAamp DNA minikit (Qiagen, Hilden, Germany). Library preparations were constructed following the manufacturer’s protocol (Illumina TruSeq DNA Nano library prep kit) and then sequenced using the Illumina HiSeq PE150 platform to generate 350-bp paired-end reads. The total number of reads after filtering and trimming for the SM7059 library was 11,249,876. For read trimming, raw data were processed, including removal of bases if the quality was below 20, removal of reads with a length below 50 bp, and removal of adapter contamination and duplicated reads, using Trimmomatic version 0.32 (3). Trimmed sequencing reads were then assembled *de novo* using the SPAdes pipeline version 3.9.0 (4), with default settings. The Prokka version 1.12 software was used to predict gene models (5). Then, all gene models were subjected to a BLAST search against the KEGG (http://www.genome.jp/kegg/) (6) and COG (http://www.Ncbi.Nlm.Nih.gov/COG) (7) databases to perform functional annotation. The assembled sequence contains 4,189 protein-coding genes, 6 ribosomal RNAs (rRNAs), and 75 transfer RNAs (tRNAs). The chromosome comprises 4,646,251 bp, with a G+C content of 66.41%. A total of 75 contigs were generated, and the *N*_50_ contig size was 204,159 bp. Multilocus sequence typing (MLST) was performed according to the reference method (8). Briefly, the seven housekeeping genes of S. maltophilia were PCR amplified and sequenced; the locus included *atpD, gapA, guaA, mutM, nuoD, ppsA,* and *recA*. The result showed that S. maltophilia SM7059 belongs to sequence type 115 (ST115; MLST 2.0 [http://www.genomicepidemiology.org]). Notably, the genes *cc4* and *cccA*, encoding cytochrome *c*_4_ and cytochrome *c*_550_, respectively, were annotated in the sequence of SM7059, which indicates the reason that strain SM7059 has a positive result in the oxidase test (9).

In conclusion, the genome sequence and the results of bioinformatics analysis of this S. maltophilia strain provide a basis for understanding the expression of oxidase in this strain. Complete genome sequencing may provide valuable information for the phenotypes of clinical species.

### Data availability.

This genome sequence has been deposited in GenBank under the accession number QDID00000000. Raw sequence reads have been deposited in the NCBI Sequence Read Archive under the accession number SRR8776274.
